# Genome-wide analysis of the bHLH gene family in Chinese jujube (*Ziziphus jujuba* Mill.) and wild jujube

**DOI:** 10.1186/s12864-019-5936-2

**Published:** 2019-07-10

**Authors:** Hongtai Li, Weilin Gao, Chaoling Xue, Yao Zhang, Zhiguo Liu, Yu Zhang, Xianwei Meng, Mengjun Liu, Jin Zhao

**Affiliations:** 1 College of Life Science, Hebei Agricultural University, Baoding, China; 2 Hebei Key Laboratory of Plant Physiology and Molecular Pathology, Hebei Agricultural University, Baoding, China; 3 Research Center of Chinese Jujube, Hebei Agricultural University, Baoding, China; 4 College of Forestry, Hebei Agricultural University, Baoding, China

**Keywords:** *ZjbHLHs*, Chinese jujube, Tissue-specific expression, Flower and fruit development, Phytoplasma, Protein-protein interaction

## Abstract

**Background:**

The bHLH (basic helix-loop-helix) transcription factor is one of the largest families of transcription factors in plants, containing a large number of members with diverse functions. Chinese jujube (*Ziziphus jujuba* Mill.) is the species with the highest economic value in the family Rhamnaceae. However, the characteristics of the bHLH family in the jujube genome are still unclear. Hence, *ZjbHLHs* were first searched at a genome-wide level, their expression levels under various conditions were investigated systematically, and their protein-protein interaction networks were predicted.

**Results:**

We identified 92 *ZjbHLHs* in the jujube genome, and these genes were classified into 16 classes according to bHLH domains. Ten ZjbHLHs with atypical bHLH domains were found. Seventy *ZjbHLHs* were mapped to but not evenly distributed on 12 pseudo- chromosomes. The domain sequences among *ZjbHLH*s were highly conserved, and their conserved residues were also identified. The tissue-specific expression of 37 *ZjbHLH* genes in jujube and wild jujube showed diverse patterns, revealing that these genes likely perform multiple functions. Many *ZjbHLH* genes were screened and found to be involved in flower and fruit development, especially in earlier developmental stages. A few genes responsive to phytoplasma invasion were also verified. Based on protein-protein interaction prediction and homology comparison, protein-protein interaction networks composed of 92 ZjbHLHs were also established.

**Conclusions:**

This study provides a comprehensive bioinformatics analysis of 92 identified *ZjbHLH* genes. We explored their expression patterns in various tissues, the flowering process, and fruit ripening and under phytoplasma stress. The protein-protein interaction networks of ZjbHLHs provide valuable clues toward further studies of their biological functions.

**Electronic supplementary material:**

The online version of this article (10.1186/s12864-019-5936-2) contains supplementary material, which is available to authorized users.

## Background

Transcription factors (TFs) are important regulatory factors in eukaryotes that interact with cis-elements to regulate the expression of specific genes in response to environmental stresses [1]. According to the sequence of arginine and lysine residues in the DNA binding region, the TFs of higher plants can be divided into four categories: zinc finger [2], helix-turn-helix (HTH) [3], basic leucine zipper (bZIP) [4], and helix-loop-helix (HLH) [5].

The basic helix-loop-helix (bHLH) family is one of the largest TF families in plants [6]. The bHLH domain is composed of approximately 50–60 conserved amino acid sequences and contains two functional regions: one is the basic amino acid region with a length of approximately 15 amino acids at the N-terminal, and the other is the HLH region at the C-terminal [7]. The basic region of approximately 15 amino acids is responsible for binding to the E-box (CANNTG) element. Studies have shown that two helices of the same transcription factor or different transcription factors interact to form homologous or heterologous dimers, which can combine with different parts of the gene promoter to regulate the target gene [8]. Moreover, some atypical bHLHs with a less basic region that is critical for DNA binding were further identified and characterized in Arabidopsis [9–11].

As an increasing number of genome sequences are being released, a variety of bHLH superfamily genes have been identified and analyzed in a wide range of plant species, such as Arabidopsis [12], pear [13], peach [1], apple [14], grape [15] and cotton [16]. Furthermore, the functions of many bHLH proteins in plants have been studied in detail. bHLHs play various roles in plant development [16–18], signal transduction [8], tolerance [19–21] and secondary metabolite production [22]. Identification of *bHLHs* at a genome-wide level is the first step toward further studies into their biological functions. However, the bHLH transcription factors in Chinese jujube (*Ziziphus jujuba* Mill.) have not been reported before and the related functions of *ZjbHLHs* are still unknown.

Chinese jujube is a species with high economic value in the family Rhamnaceae and is also one of the most representative national fruit trees in China. Wild jujube, the wild relative species of Chinese jujube, usually has smaller trees and fruits than the Chinese jujube. Both jujube and wild jujube trees have many agronomic advantages, such as early fruit production, long flowering season and high tolerance to various biotic and abiotic stresses. And the bHLH gene family has a variety of functions such as flower and fruit development and is necessary for the normal growth and development in many plants [8, 16–22]. Compared with other TFs, bHLHs are involved in more reaction pathways and acted as some co-regulators on gene expression together with many other proteins. Therefore, we want to figure out the functions of this gene family in jujube. The jujube genome database [23, 24] provided the possibilities and resources for searching the crucial gene families related to its biological characteristics at the genome level. For the wide and diverse biological roles of *bHLHs* in plant development, the gene number, classification, and gene structure of this family in the jujube genome and their expression under various conditions in jujube and wild jujube were systematically analyzed in this study, and the protein-protein interaction networks were also predicted. The results provide valuable clues for further revealing the functions of this family in jujube growth and development.

## Results

### Identification of *ZjbHLHs*

A total of 92 nonredundant putative *bHLH* transcripts (Table 1) were identified in the jujube genome sequence (https://www.ncbi.nlm.nih.gov/genome/?term=jujube) [23]. To verify the reliability of each sequence, 92 protein sequences were analyzed using the online CD-search and SMART tools, and 82 bHLH proteins were found to have a typical bHLH structure except for the 10 bHLH proteins with an atypical bHLH domain (Additional file 1: Figure S1). They were named from *ZjbHLH1* to *ZjbHLH92* according to their gene structure and motifs, and the *ZjbHLH2*, *7*, and *54* sequences were new genes with no information in NCBI. The ORF length for *ZjbHLH* genes ranged from 285 bp (*ZjbHLH59*) to 2676 bp (*ZjbHLH57*), and the genes encoded proteins ranging from 94 (*ZjbHLH59*) to 891 (*ZjbHLH57*) amino acids (aa) in length, with predicted pIs ranging from 4.62 (*ZjbHLH1*) to 9.86 (*ZjbHLH41*). The proteins with an isoelectric point of less than 7 accounted for 66% of the total, which means that most of the *ZjbHLH* genes were weakly acidic. Based on their physical and chemical properties, the family members have different characteristics, indicating that they likely have multiple functions.

**Table 1:**
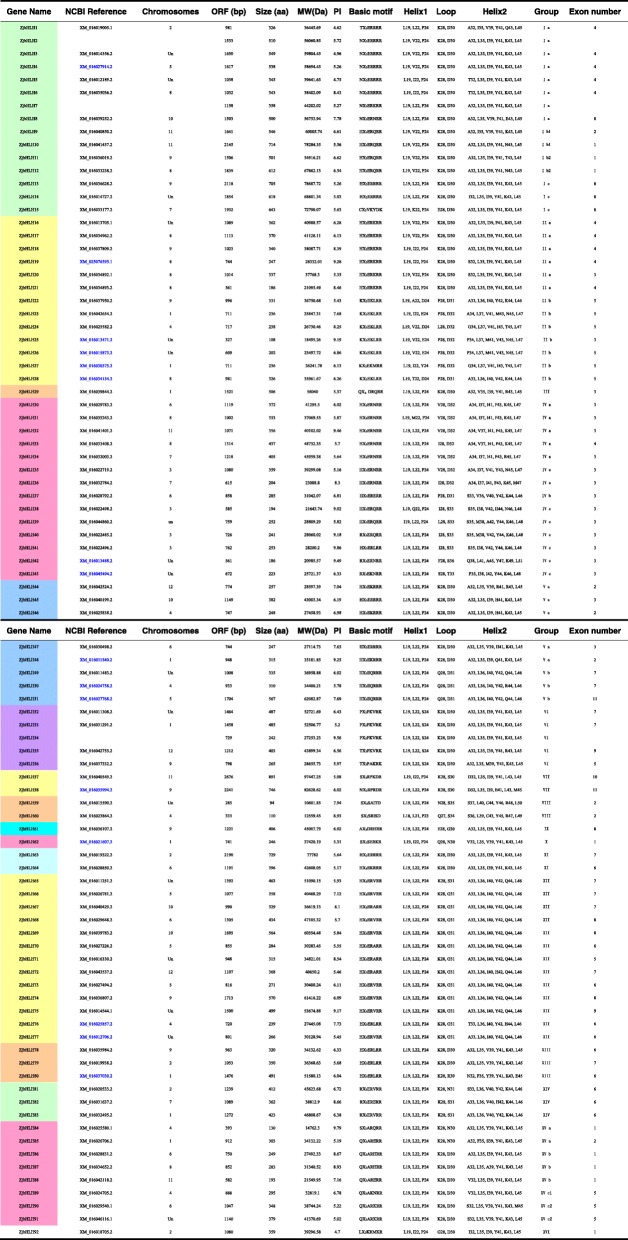
The information of bHLH gene family in Chinese jujube

Previous genome evolution studies showed that Chinese jujube is closely related to species of the Rosaceae family [23], so the number of bHLHs from three Rosaceae species (apple, pear and peach), grape, cotton and Arabidopsis were compared with ZjbHLHs (Additional file 2: Table S1). There were similar gene numbers in the bHLH family of jujube and peach, and this result was consistent with a previous study on the MADS-box family [25]. Compared with the number of *bHLH* genes found in other plant species, the number of *bHLH* genes found in jujube and peach was lower. The gene numbers may be related to evolutionary differences, genome replication, or the genome size of these plants [1].

### Phylogenetic analysis of ZjbHLHs

**Fig. 1:**
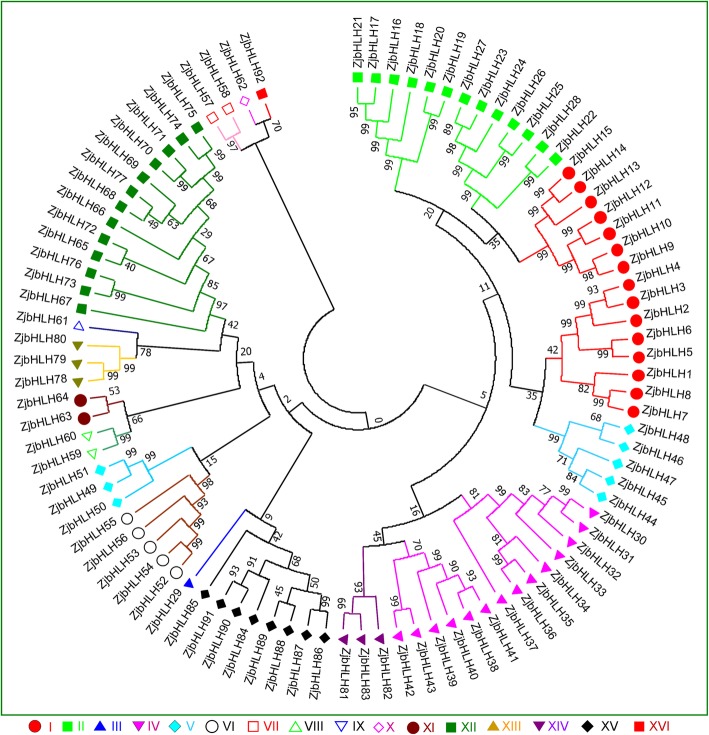
The phylogenetic analysis of ZjbHLHs. The NJ tree was constructed from the protein sequences of ZjbHLHs using MEGA7 with 1000 bootstrap copies

A phylogenetic tree of the ZjbHLH proteins was constructed by aligning multiple domain sequences (Fig. 1). A phylogenetic tree of *bHLH* genes of Arabidopsis and jujube was established, and *ZjbHLH*s were divided into 16 categories (Fig. 1). And the bHLH phylogenetic trees of peach [1] and jujube (Additional file 3: Figure S2) was constructed, which further verified the above classification result. The *bHLH* genes of the six species (jujube, apple, pear, peach, grape, cotton and Arabidopsis) showed a mixed pattern on the evolutionary tree (Additional file 4: Figure S3). These results indicated that the *bHLH* genes were present before the divergence of various plant species and then expanded in each species independently.

### Multiple sequence alignment and conserved motifs in ZjbHLHs

**Fig. 2:**
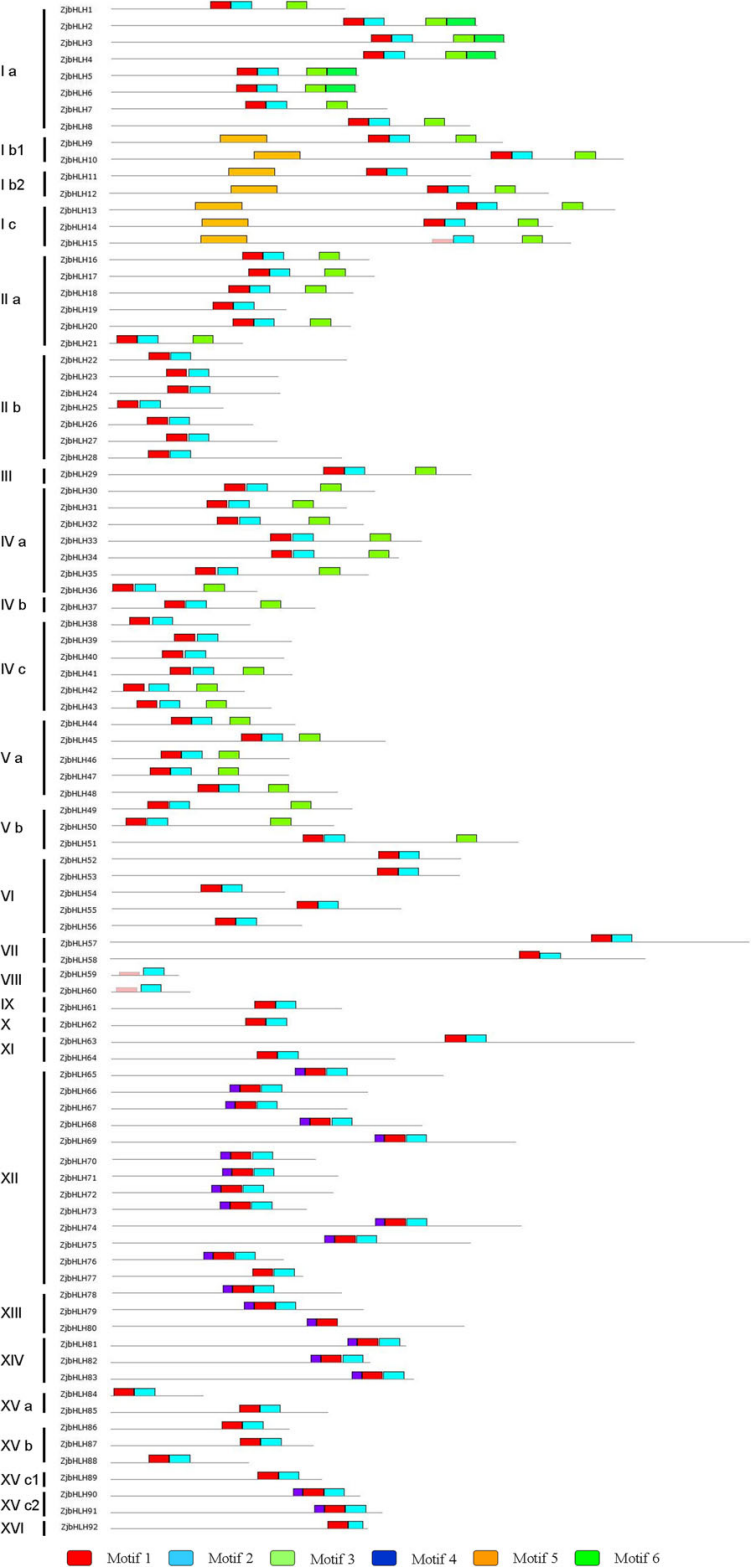
Conserved motifs of ZjbHLH proteins. The motifs in ZjbHLHs were identified by using Multiple Em for Motif Elicitation (MEME). In these ZjbHLH proteins, six conserved motifs were identified and displayed in different colors

Comparison of multiple sequences showed that most of the *ZjbHLH* genes had the same conserved domain structure except for the VI, VII and VIII *ZjbHLH* genes (Fig. 2). The domain sequences in the ZjbHLH gene family were highly conserved (Additional file 1: Figure S1). Five residues (His-1, Glu-5, Arg-6, Arg-8, and Arg-9), three residues (Leu-19, Leu-22, and Pro-24), two residues (Lys-28 and Asp-30) and six residues (Ala-32, Leu-35, Ile-39, Tyr-41, Lys-43, and Leu-45) made up the basic region, the first helix region, the loop region and the second helix region, respectively. There were 6 motifs among ZjbHLHs*,* and proteins in the same group had similar numbers and types of motifs (Additional file 5: Figure S4). The bHLH domains Motif 1 and Motif 2 were highly conserved among the 92 proteins (Additional file 6: Figure S5), and only ten (*ZjbHLH15, ZjbHLH52*~*ZjbHLH60*) of them contained variations. *ZjbHLH59* and *ZjbHLH60* were identified as atypical *bHLH* genes by the Conserved Domain Search Service (CD Search) [26].

### The chromosomal location and gene structure of *ZjbHLHs*

**Fig. 3:**
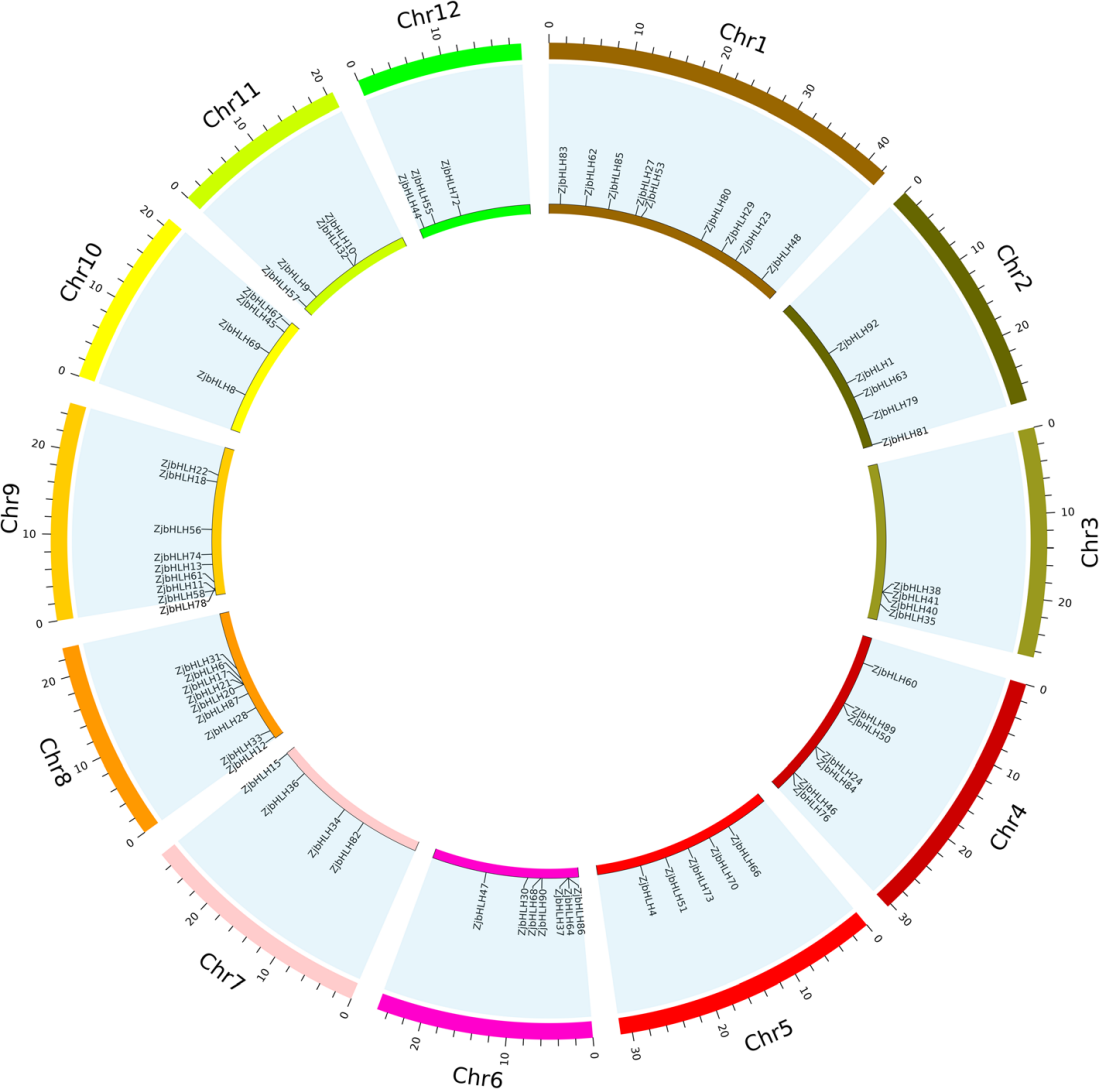
The chromosomal location of 70 *ZjbHLH* genes*.* Genes are mapped to jujube chromosomes by the Circos tool. The chromosomes of jujube are arranged in a circle

Of the 92 *ZjbHLH* genes, 70 were mapped to 12 pseudo-chromosomes in the jujube genome (Fig. 3), 17 genes were located on 12 scaffolds, and 5 genes were uncommented. *ZjbHLHs* were not evenly distributed across the 12 chromosomes. Nine *ZjbHLH* genes (9.8%) were on Chr. 1, 8, and 9, and 7 *ZjbHLHs* (7.6%) were located on Chr. 4 and 6. Furthermore, some *ZjbHLHs* concentrated on part of the chromosome, and some relatively high-density *bHLH* genes were observed in some chromosomal regions. Some genes were tightly packed into clusters to form tandem repeats (*ZjbHLH27* and *53*; *ZjbHLH38, 40* and *41*; *ZjbHLH64* and *37*; *ZjbHLH17, 20* and *21*). A previous study analyzed repeated events in rice and Arabidopsis [27], indicating that some *bHLH* subfamily members are most likely derived from repetitive events.

**Fig. 4:**
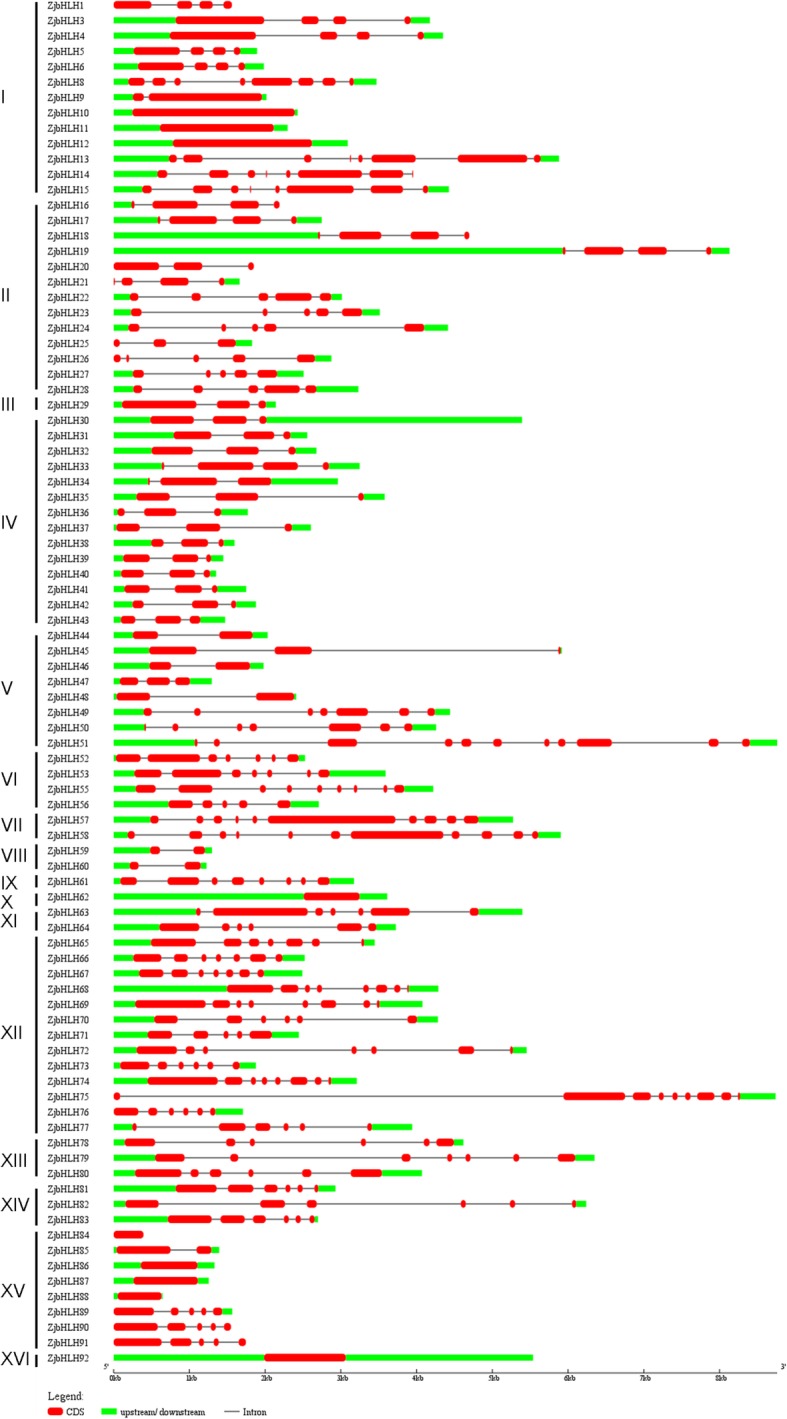
The gene structure of *ZjbHLHs* in Chinese jujube. Introns and exons are represented by black lines and red boxes respectively and upstream/downstream are represented by green boxes

Additionally, the gene structure was highly conserved within each group (Fig. 4), except for the 3 uncommented *ZjbHLHs* (2, 7 and 54). We found that Group VI, VII, IX, XI, XII, XIII, and XIV genes contained more introns and were more complicated than genes in the other groups (Fig. 4).

### Expression patterns of *ZjbHLHs* in various tissues/organs

**Fig. 5:**
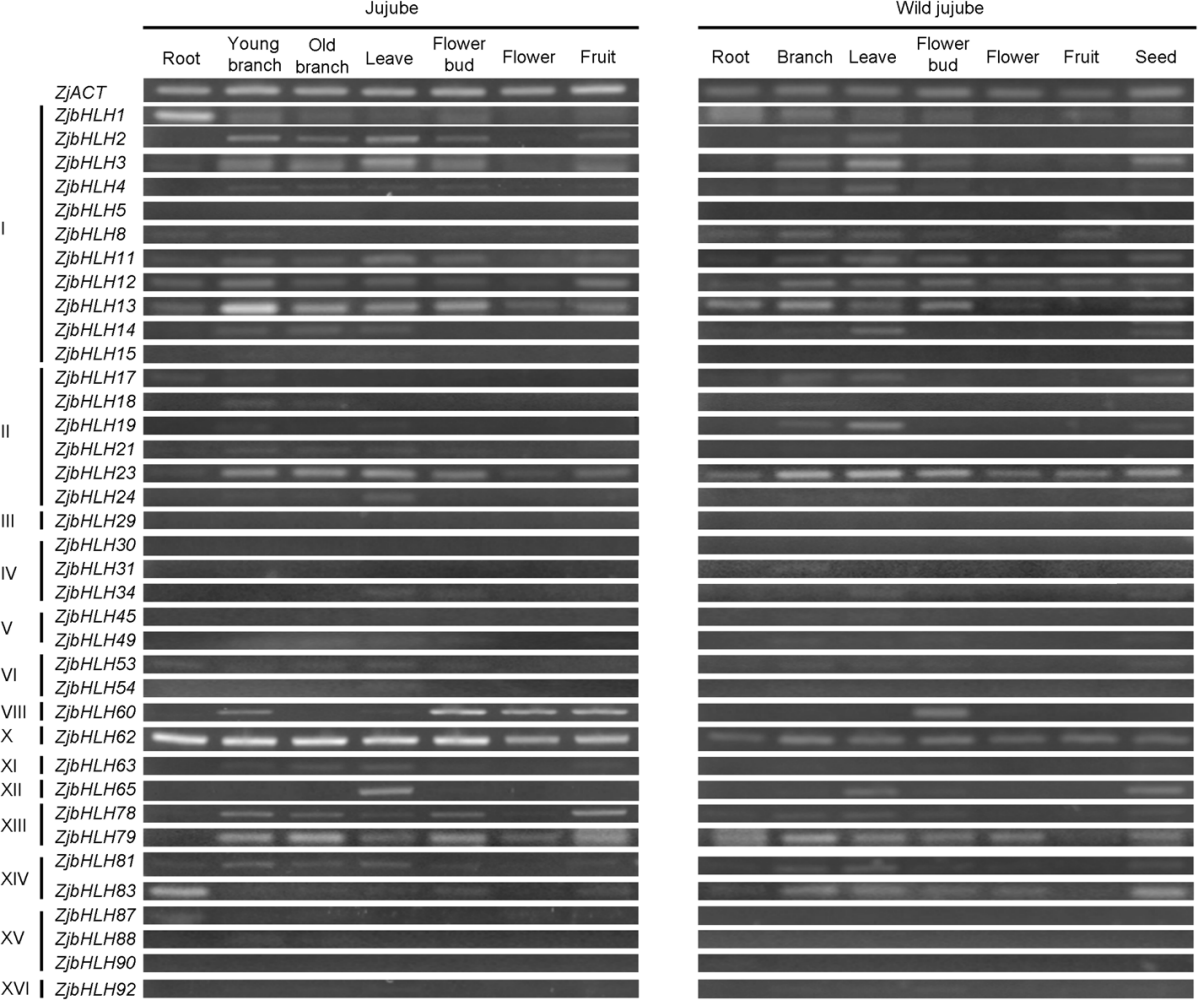
Expression patterns of 37 *ZjbHLH* genes in seven tissues of jujube and wild type jujube by RT-PCR. *ZjACT* was used as an internal control. Left: jujube, from left to right: root, young branch, old branch, leaf, flower bud, flower, and fruit. Right: wild jujube, from left to right: root, branch, leaf, flower bud, flower, fruit and seed

To explore the tissue-specific expression of *ZjbHLHs*, their expression patterns were determined in various tissues by semiquantitative PCR. The expression patterns of most examined *ZjbHLHs* were similar in jujube and wild jujube (Fig. 5), except for 8 *ZjbHLHs* (17, 21, 54, 60, 63, 79, 83, and 87). Some genes were mainly expressed in vegetative organs (*ZjbHLH1, 2, 3, 4, 11, 63, 65, 81, 83, and 87*) or reproductive organs (*ZjbHLH60*). In particular, *ZibHLH62* was stably expressed in various organs of both jujube and wild jujube and can be used as a housekeeping gene. These results showed that most of the *ZjbHLHs* had diverse tissue-specific expression patterns, indicating that they play multiple roles in various organs.

In addition, the expression of *ZjbHLH8* and *19* genes in the branches and leaves of jujube was significantly weaker than that in wild jujube. This differential expression indicated that some *ZjbHLHs* may have different functions between jujube and wild jujube.

### *ZjbHLHs* involved in flower and fruit development

**Fig. 6:**
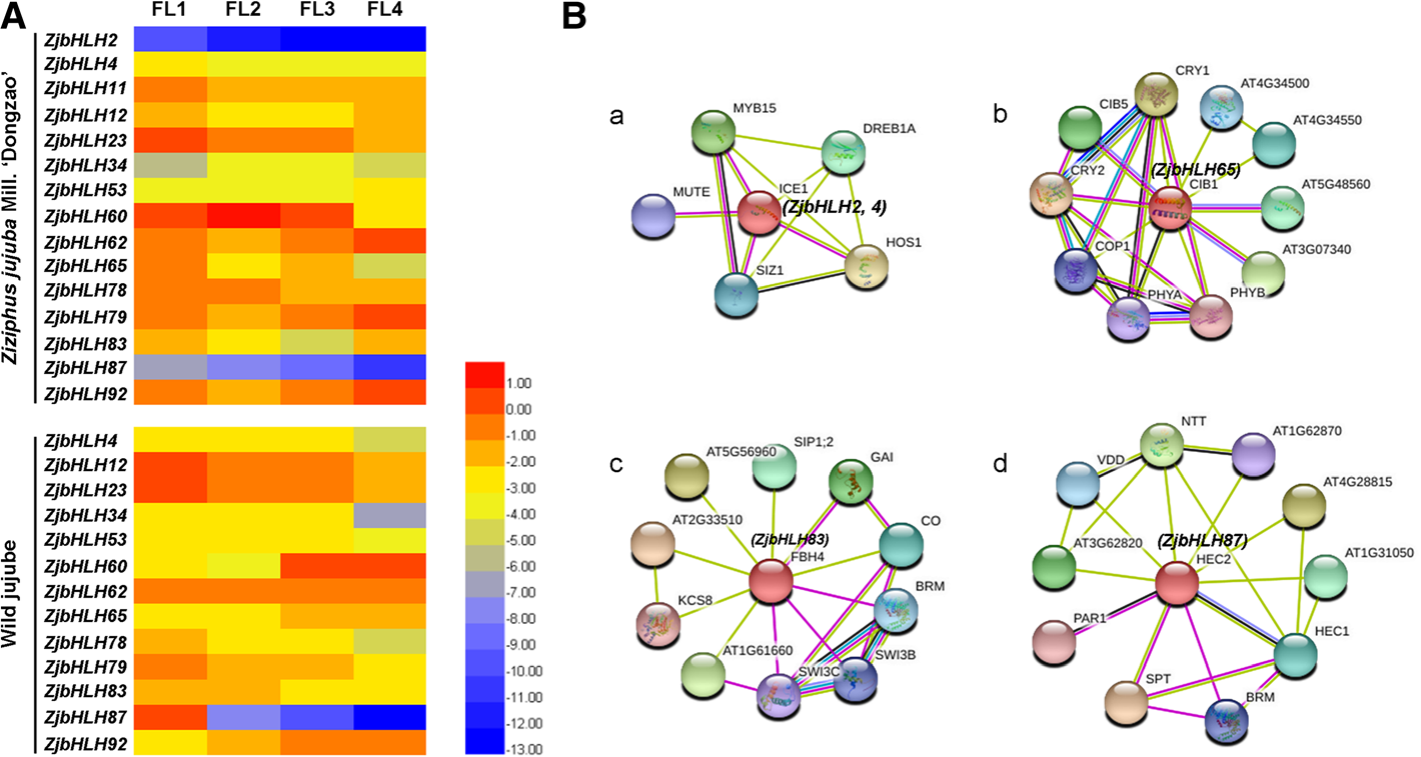
**a** Heat maps of the relative expression of *ZjbHLH* genes during flower development. FL1, bud emergence stage; FL2, inflorescence emergence stage; FL3, yellow bud stage; FL4, petal spread stage. Scaled log2 expression values based on qRT-PCR data are shown from blue to red, indicating low to high expression. **b** The protein-protein interaction analysis of four ZjbHLHs by STRING database

Based on the tissue-specific expression, the expression of *ZjbHLHs* was further detected at four floral developmental stages (Fig. 6a). Among them, *ZjbHLH62* and *ZjbHLH53* were expressed stably at the four stages in jujube and wild jujube. The expression levels of *ZjbHLH4, 12, 23, 78* and *87* genes decreased gradually with flower development, and *ZjbHLH92* had a high expression level at the later stages in both jujube and wild jujube. It is remarkable that the expressions of *ZjbHLH4*, *12*, *34*, *60*, *62*, *78*, *79* and *83* genes in four stages showed opposite trends between jujube and wild jujube and four genes (*ZjbHLH4*, *12*, *60* and *78*) showing significantly different expression were screened out (Additional file 9: Figure S6), indicating that they may perform different functions during flower development in jujube and its wild-type species. Through protein-protein interaction prediction and homology comparison, it is predicted that *ZjbHLH2, 4, 65, 83,* and *87* genes have crucial functions during flower development (Fig. 6b).

**Fig. 7:**
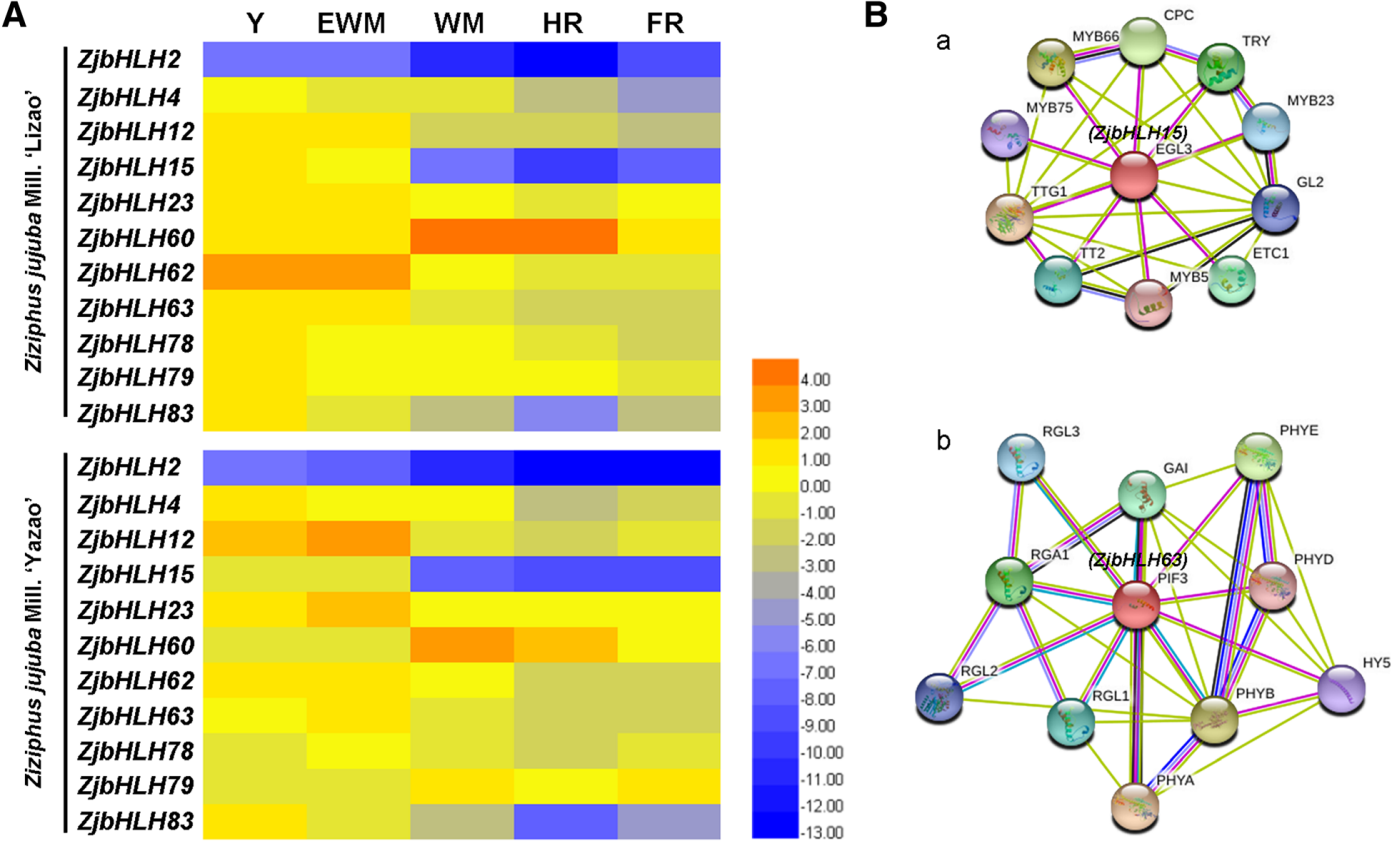
**a** Heat maps of the relative expression of *ZjbHLH* genes during fruit ripening in ‘Lizao’ and ‘Yazao’. Y, young fruit; EWM, early white mature fruit; WM, white mature fruit; HR, half-red fruit; FR, full red fruit. Scaled log2 expression values based on qRT-PCR data are shown from blue to red, indicating low to high expression. **b** The protein-protein interaction analysis of two ZjbHLHs by STRING database

During jujube fruit development, some genes (*ZjbHLH2, 4, 12, 15, 23, 62, 63, 78* and *83*) were highly expressed at the first two stages and then significantly decreased at later stages (Fig. 7a). However, *ZjbHLH60* was mainly expressed at the late stages. The protein interaction prediction and homology comparison also indicated that *ZjbHLH15* and *63* might play important roles in fruit development (Fig. 7b). The above results indicated that some *ZjbHLHs* were truly involved in jujube flower and fruit development.

### *ZjbHLHs* participated in jujube-phytoplasma interactions

**Fig. 8:**
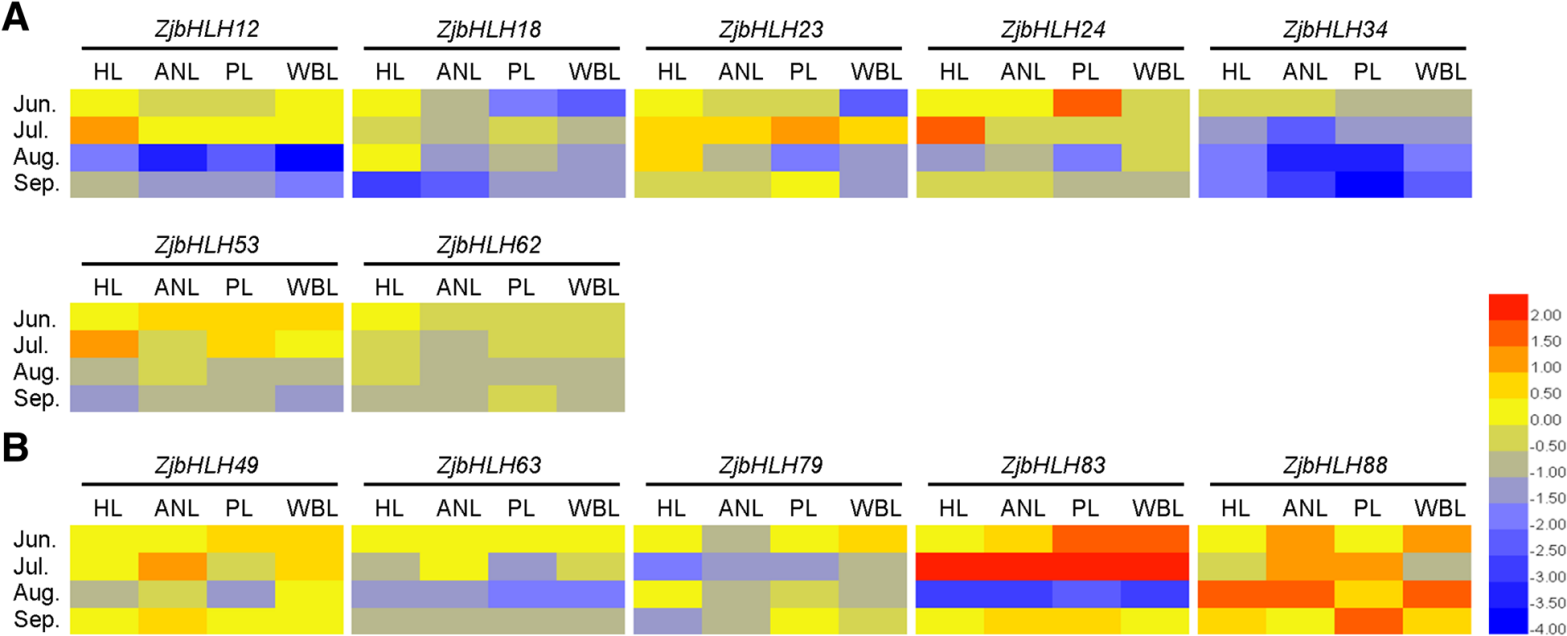
Heat maps of the relative expression of *ZjbHLH* genes under phytoplasma stress. **a** The gene expression in diseased leaves was significantly lower than that in healthy leaves. **b** The gene expression in diseased leaves was significantly higher than that in healthy leaves. Scaled log2 expression values based on qRT-PCR data are shown from blue to red, indicating low to high expression, respectively

JWB caused by phytoplasma is a destructive disease in jujube production. Since *bHLH* genes have multiple functions in plants, whether they participate in jujube-phytoplasma interactions remains unclear. Hence, their expression changes were investigated in jujube under phytoplasma stress. Among the 23 *ZjbHLH* genes detected, the expression of *ZjbHLH12, 18, 23, 24, 34, 53,* and *62* genes in diseased leaves was significantly lower than the expression in healthy leaves (Fig. 8a). *ZjbHLH49, 63, 79, 83,* and *88* genes were highly expressed in diseased leaves (Fig. 8b). These results suggested that some *ZjbHLHs* participate in jujube-phytoplasma interactions.

### ZjbHLH protein-protein interaction network prediction

**Fig. 9:**
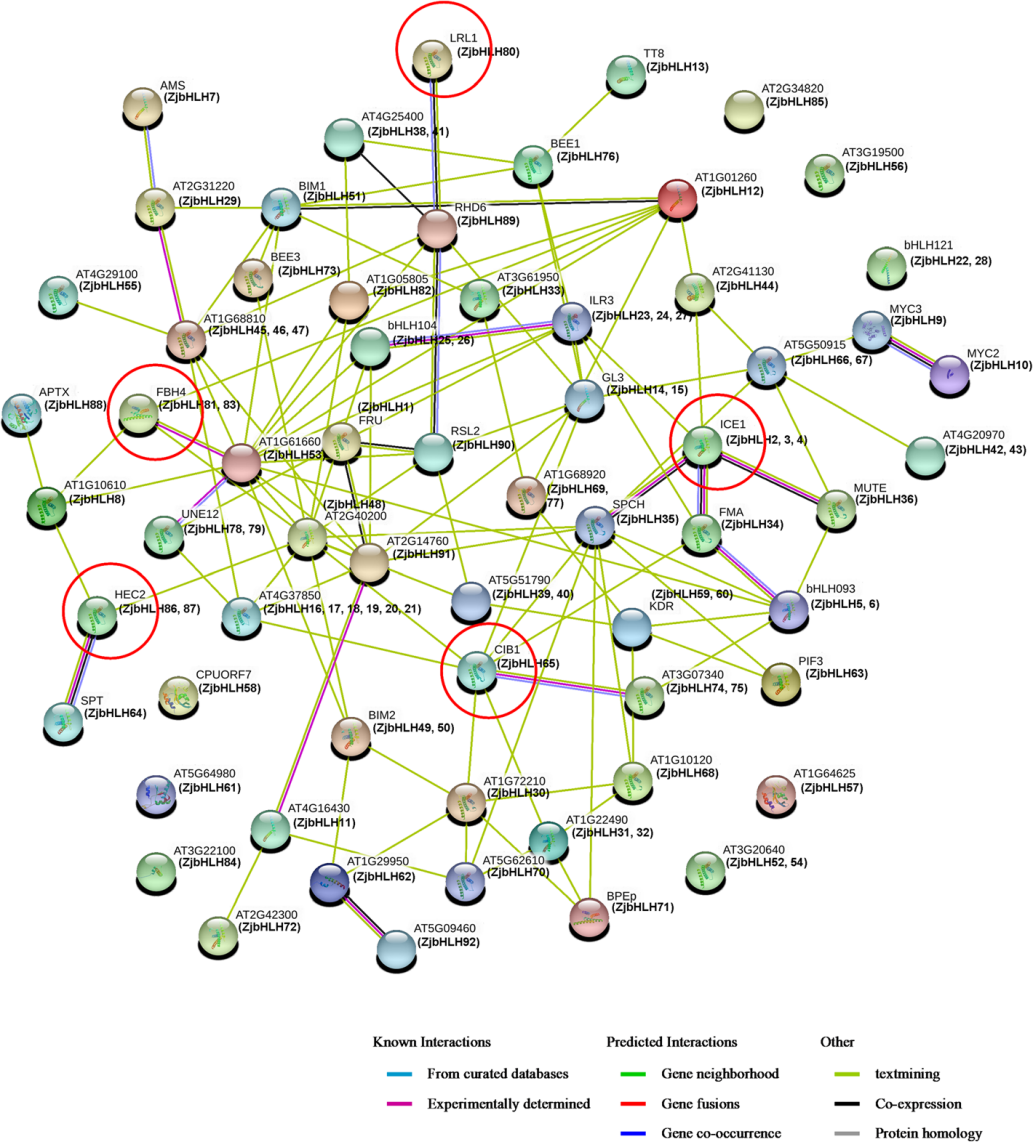
A protein-protein interaction network for ZjbHLHs based on their orthologs in Arabidopsis. This network was predicted by online software STRING. ZjbHLH proteins were shown in brackets with Arabidopsis orthologs

Based on the orthologs in Arabidopsis (Additional file 7: Table S2), it was predicted by STRING that many ZjbHLH proteins interacted with each other (Fig. 9), which is in accord with previous reports that the binding activity of bHLH proteins depends upon the formation of homodimers or heterodimers among bHLH proteins [28, 29]. Overall, several important interactions were predicted in Fig. 9. Both FBH4 (homolog of ZjbHLH81 and 83) and CIB1 (homolog of ZjbHLH65) were involved in the regulation of flowering time [30, 31], and HEC (homolog of ZjbHLH86 and 87) could interact with SPT (homolog of ZjbHLH64) to jointly regulate pistil development by regulating cytokinins and other hormones [32]. ICE1 (homolog of ZjbHLH2, 3, and 4) could interact with FMA (homolog of ZjbHLH34), SPCH (homolog of ZjbHLH35) and MUTE (homolog of ZjbHLH36) could regulate stomatal differentiation [33]. Moreover, ICE1 also regulated lateral bud growth and plant stress response [34, 35], and LRL1 (homolog of ZjbHLH80), RHD6 (homolog of ZjbHLH89) and RSL2 (homolog of ZjbHLH90) were involved in the regulation of root hair development [36, 37]. These results further proved the functional diversity of *ZjbHLH* genes. In addition, we also found that the functions of those genes contained more introns were mostly related to flower and root development (Additional file 7: Table S2). The predicted network provides some useful clues for functional studies, further experimental evidences should be needed.

## Discussion

In this study, a total of 92 *bHLH* genes were identified in the jujube genome. Based on phylogenetic analysis, intron-exon gene structure, conserved protein motifs and amino acid physical and chemical property prediction, these ZjbHLHs were divided into 16 categories; most sequences have the same conservative sequence except for the VI, VII, and VIII groups. Among them, there are 10 atypical sequences, and this trait has also been demonstrated in other plants [25]. Furthermore, our BLAST results strongly supported our classifications of the ZjbHLHs, and detailed information about these orthologs was also summarized (Additional file 7: Table S2).

Many ZjbHLH proteins are involved in jujube flower development. *ZjbHLH2* and *ZjbHLH4* were expressed at higher levels at earlier flower development stages. These two genes belong to the ICE1 branch, which can regulate lateral bud growth [35]. A similar function was also confirmed by homologous protein interactions (Fig. 6B-a). ICE1 can interact with the HOS1 protein, which is an important regulator of flowering time [38]. ZjbHLH65, 83 and 87 proteins were homologous to CIB4, FBH4 and HEC2, respectively, and were three key regulatory factors in flower development (Fig. 6B-b, c, d). *ZjbHLH83* is the homologous gene of FBH4 (At2g42280). Overexpression of FBH4 in Arabidopsis drastically elevated CO expression and caused early flowering regardless of the photoperiod [30]. Here, *ZjbHLH83* showed high expression at earlier flower development stages (Fig. 6a) and was also predicted to interact with CO (Fig. 6B-c). In addition, a series of CIB genes (CIB1, 2, 3, 4, and 5) in Arabidopsis can activate FT transcription by interacting with CRY2 protein and mediate the regulation of flowering time [31]. There are also a series of CIB homologous genes in jujube, namely, *ZjbHLH65* (homologs of CIB1), *ZjbHLH74* and *75* (homologs of CIB3), *ZjbHLH68* (homologs of CIB4), and *ZjbHLH69* (homologs of CIL1). Since CIB genes have been proven to be conserved among plants [31], they are likely to have a similar function in flower development.

In fruit development, the expression patterns of most *ZjbHLH* genes detected in the two cultivars were in line with each other, which indicated that their functions in fruit development might be conserved among jujube varieties. *ZjbHLH63* expression was significantly higher at the early stage of fruit development (Fig. 7a), which is the period of fruit enlargement. ZjbHLH63 is the homolog of AtPIF3 (Fig. 9), a key factor affecting light morphogenesis [39]. The homologous comparison and protein interaction prediction (Fig. 7B-b) also indicated that it might be involved in fruit enlargement. In addition, ZjbHLH15 homologous protein in Arabidopsis was predicted to be involved in fruit anthocyanin synthesis (Fig. 7B-a), but in this study, its expression decreased significantly at the fruit coloring stages (Fig. 7a). Therefore, we hypothesized that jujube fruit color changes might not correlate with the accumulation of anthocyanin.

In addition, the expression patterns of some bHLH genes in jujube and wild jujube were not the same, indicating that *ZjbHLH* genes participate in different regulation pathways between jujube and wild jujube, especially in flower development. Further studies are needed to elucidate the detailed interaction network of the growth and development of jujube and wild jujube.

## Conclusions

This study described the bHLH gene family of Chinese jujube at the genome level. Their gene structure, chromosomal distribution, phylogenetic relationship, and tissue-specific expression patterns were presented. Ten ZjbHLHs with atypical bHLH domains were identified. Many *ZjbHLH* genes were confirmed to involve in flower and fruit development and responsive to phytoplasma stress. An integrated ZjbHLHs protein-protein interaction network was also predicted. These results are very meaningful to the future functional analysis of ZjbHLHs.

## Methods

### Plant materials

Chinese jujube and wild jujube trees used in this study are cultivated in the Experimental Station of Chinese Jujube, Hebei Agricultural University. No specific permits are required for the sample collection. They are not endangered or protected species.

Seven tissue types (roots, young branches, old branches, leaves, flower buds, flowers and young fruits) collected from three jujube trees and three wild jujube trees were used for organ-specific expression analysis. The flowers of jujube and wild jujube were used for qRT-PCR analysis. The four development stages sampled were the bud emergence stage (FL1), inflorescence emergence stage (FL2), yellow bud stage (FL3) and petal spread stage (FL4). The fruits of two jujube cultivars (‘Lizao’ and ‘Yazao’) were used to investigate the expression pattern of *ZjbHLHs*. Five developmental stages, including the young fruit stage (Y), early white mature fruit stage (EWM), white mature fruit stage (WM), half-red fruit stage (HR) and full-red fruit stage (FR), were sampled. Each treatment was collected from three biological replicates.

Three kinds of tissues representing disease symptoms of different severity of Jujube witches’ broom (JWB) disease (apparently normal leaves (ANL), phyllody leaves (PL), and witches’ broom leaves (WBL)) from diseased trees and healthy leaves (HL) from healthy trees were collected in four periods (June, July, August and September). All treatments were conducted with three biological replicates.

### Identification and protein structure analysis of ZjbHLHs

The hidden Markov model (HMM) file of the bHLH domain (PF00010) was downloaded from the Pfam database (http://pfam.xfam.org/), and HMMER 3.1b2 software was used to find the ZjbHLH protein sequences in the jujube genome [23]. To further confirm our sequences, we used the online CD-search tool (NCBI database), the SMART tool (http://smart.embl-heidelberg.de/) and the website of PlantTFDB to screen sequences. Truncated and false genes were excluded from our analysis. The number of amino acids, molecular weight, and theoretical pI of *ZjbHLH* genes were predicted by NCBI and ProtParam (https://web.expasy.org/compute_pi/). The conserved motifs of ZjbHLH proteins were detected by MEME (http://meme-suite.org/) [40].

### The chromosomal location and gene structure of *ZjbHLHs*

To determine the chromosomal location of the *ZjbHLH* genes, their gene sequences were used as query sequences in BLASTN searches against the jujube genome. Each *ZjbHLH* gene was mapped to the jujube genome according to its genome coordinates. Tandem duplications were identified as previously described [41].

The website GSDS (http://gsds.cbi.pku.edu.cn/) was used to predict the number of exons from the coding domain sequences (CDS) and DNA sequences of the *ZjbHLH* genes [42].

### Multiple sequence alignment and phylogenetic tree construction

Multiple sequence alignment was analyzed by using ClustalX2 and edited by BioEdit. A phylogenetic tree of 92 ZjbHLHs was constructed based on their conserved domains. bHLH proteins of six other species (*Arabidopsis thaliana, Prunus persica*, *Malus domestica*, *Pyrus bretschneideri, Vitis vinifera* L. and *Anemone vitifolia* Buch.) were downloaded from NCBI. MEGA 7 software and the neighbor-joining statistical method were used to construct a rooted phylogenetic tree [43, 44]. The evolutionary distances were obtained using the p-distance method, and these distances were used to estimate the number of amino acid substitutions per site. The reliability of each phylogenetic tree was established by conducting 1000 bootstrap sampling iterations.

### RNA isolation and expression analysis

Total RNA was extracted using an RNAprep Pure Plant Kit (TIANGEN) according to the manufacturer’s protocol. After genomic DNA was removed by RNase-free DNase I (TIANGEN), the RNA concentration and purity were checked on a NanoDrop2000 spectrophotometer. First-strand cDNA was synthesized by reverse transcribing 500 ng of total RNA with a FastQuant RT Super Mix Kit (TIANGEN). The cDNA was used as the template for gene expression analysis.

Gene expression was detected by semiquantitative PCR and qRT-PCR. The primers used in this study are listed in Additional file 8: Table S3. PCR products were amplified in triplicate using Bio-Rad iQ™5 with TransStart Top Green qPCR SuperMix AQ131 (TransGen Biotech, China) in 20 μL reactions. Each reaction contained 10 μL of 2 × TransStart® Top Green qPCR SuperMix, 0.4 μL each of 10 μM primers, 8.2 μL of ddH_2_O and 1 μL of cDNA. The thermal profile for RT-qPCR was as follows: preincubation for 30 s at 95 °C, followed by 40 cycles of 5 s at 95 °C, 10 s at 53–58 °C, and 10 s at 72 °C. Three biological replicates were performed for each treatment. Threshold cycle values were calculated using iCycler software, and *ZjACT* was used as an internal control [45]. Relative transcript levels were calculated according to the 2^–ΔΔCT^ method [46].

### Protein-protein interaction network prediction

Ninety-two ZjbHLH protein sequences were used as queries, and protein-protein interactions were predicted by the STRING website (https://string-db.org/). The orthologs of *Arabidopsis thaliana* were selected as references. After completing the BLAST step, the network was constructed using the highest score gene (bitscore). Finally, an interaction network among ZjbHLHs was constructed in this study.

## Additional files

Additional file 1: **Figure S1.** The multiple sequence alignment in ZjbHLH proteins. (DOC 3607 kb)

Additional file 2: **Table S1.** Number of bHLH gene family from Chinese jujube and other six species. (DOC 50 kb)

Additional file 3: **Figure S2.** The phylogenetic analysis of bHLH proteins of *Ziziphus jujuba* and *Persica prunu.* The NJ tree was constructed from the protein sequences of ZjbHLHs and PpbHLHs using MEGA7 with 1000 bootstrap copies. (DOC 483 kb)

Additional file 4: **Figure S3.** The phylogenetic analysis of bHLH proteins of *Ziziphus jujuba*, *Arabidopsis thaliana, Persica prunus, Malus domestica, Pyres bretschneideri, Vitis vinifera* and *Gossypium raimondii.* There are 16 categories in total, and I, IV, XI and XV are selected for display. (DOC 1096 kb)

Additional file 5: **Figure S4.** The amino acid sequences of 6 motifs among ZjbHLH proteins. (DOC 240 kb)

Additional file 6: **Figure S5.** The major functional domain of ZjbHLH proteins. (DOC 384 kb)

Additional file 7: **Table S2.** Summary information for 92 ZjbHLH proteins in STRING database. (XLS 95 kb)

Additional file 8: **Table S3.** The primers of *ZjbHLH* genes used in this study. (DOC 142 kb)

Additional file 9: **Figure S6.** Expression patterns of four *ZjbHLH* genes in flower development stage of jujube and wild jujube. FL1, bud emergence stage; FL2, inflorescence emergence stage; FL3, yellow bud stage; FL4, petal spread stage. The expression levels of eight treatments (four development stages in jujube and wild jujube, respectively) were compared and analyzed either between different stages of the same species or between different species of the same stage. All statistical analyses were performed with SPSS software 17.0. Duncan’s multiple range tests were used to assess differences between treatments. Different letters mean significant difference at 0.05 levels between the corresponding treatments. (DOC 319 kb)

## Data Availability

All data and materials are presented in the main manuscript and additional supporting file.

## Abbreviations

ANL: Apparently normal leaves
bHLH: Basic helix-loop-helix
CDS: Coding domain sequences
EWM: Early white mature fruit stage
FR: Full-red fruit stage
HL: Healthy leaves
HMM: Hidden Markov model
HR: Half-red fruit stage
JWB: Jujube witches’ broom disease
PL: Phyllody leaves
qRT-PCR: Quantitative real-time PCR
TFs: Transcription factors
WBL: Witches’-broom leaves
white WM: mature fruit stage
Y: Young fruit stage
