# Supplementary figures and images for: Genome-wide analysis of the bHLH gene family in Chinese jujube (Ziziphus jujuba Mill.) and wild jujube
Source: BMC Genomics. 2019 Jul 10;20:568. doi: 10.1186/s12864-019-5936-2 (PMC6617894; doi:10.1186/s12864-019-5936-2)

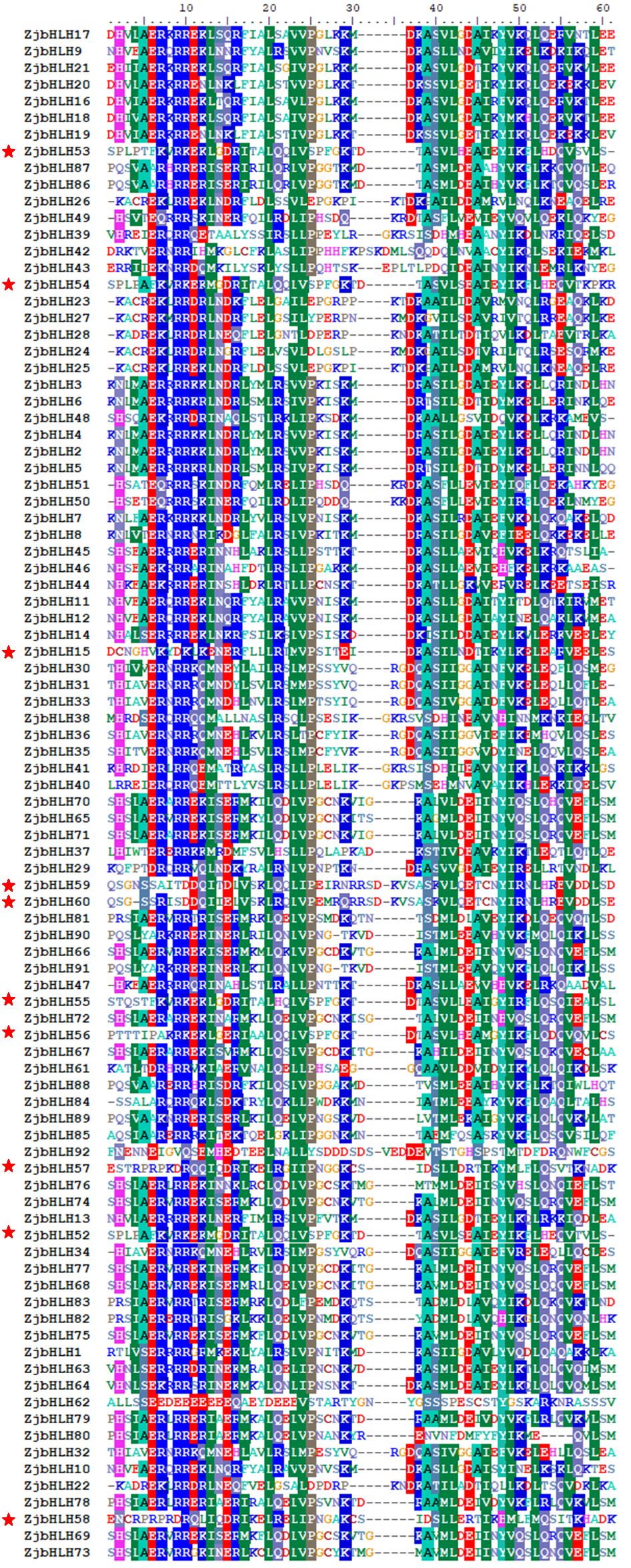

Supplement: Supplementary file 1 — Figure S1. The multiple sequence alignment in ZjbHLH proteins. (DOC 3607 kb) [file 12864_2019_5936_MOESM1_ESM.doc]

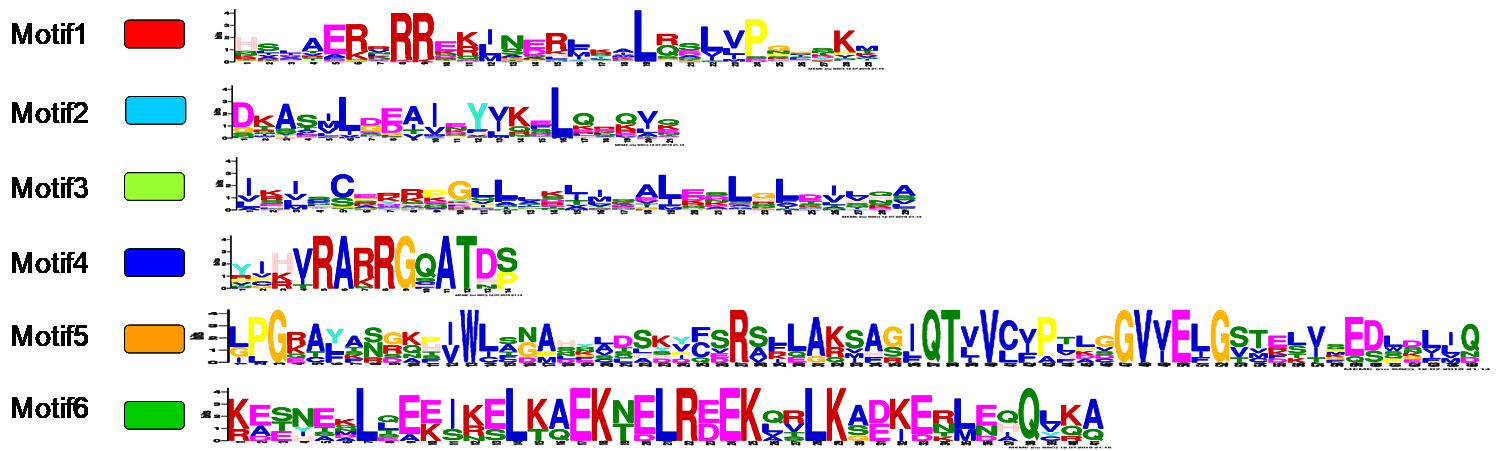

Supplement: Supplementary file 5 — Figure S4. The amino acid sequences of 6 motifs among ZjbHLH proteins. (DOC 240 kb) [file 12864_2019_5936_MOESM5_ESM.doc]

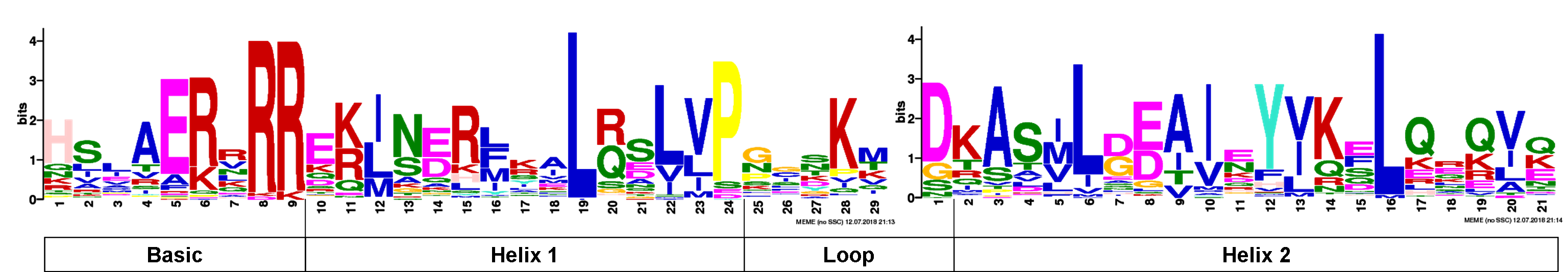

Supplement: Supplementary file 6 — Figure S5. The major functional domain of ZjbHLH proteins. (DOC 384 kb) [file 12864_2019_5936_MOESM6_ESM.doc]

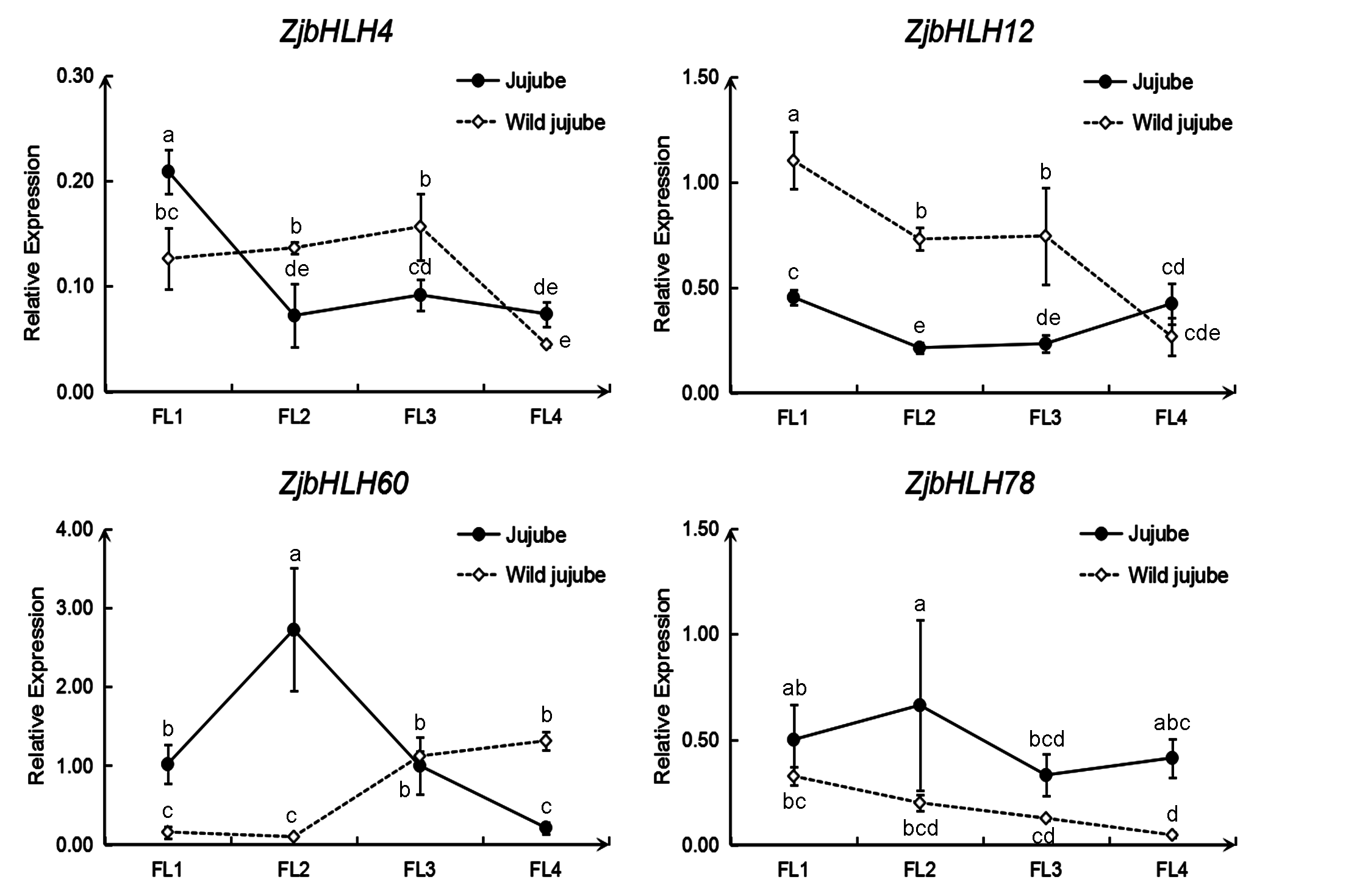

Supplement: Supplementary file 9 — Figure S6. Expression patterns of four ZjbHLH genes in flower development stage of jujube and wild jujube. FL1, bud emergence stage; FL2, inflorescence emergence stage; FL3, yellow bud stage; FL4, petal spread stage. The expression levels of eight treatments (four development stages in jujube and wild jujube, respectively) were compared and analyzed either between different stages of the same species or between different species of the same stage. All statistical analyses were performed with SPSS software 17.0. Duncan’s multiple range tests were used to assess differences between treatments. Different letters mean significant difference at 0.05 levels between the corresponding treatments. (DOC 319 kb) [file 12864_2019_5936_MOESM9_ESM.doc]
