# Supplementary material for: Genome-wide analysis of the bHLH gene family in Chinese jujube (Ziziphus jujuba Mill.) and wild jujube
Source: BMC Genomics. 2019 Jul 10;20:568. doi: 10.1186/s12864-019-5936-2 (PMC6617894; doi:10.1186/s12864-019-5936-2)
Supplement: Supplementary file 2 — Table S1. Number of bHLH gene family from Chinese jujube and other six species. (DOC 50 kb) [file 12864_2019_5936_MOESM2_ESM.doc]

| **Group** | **Gene number** | | | | | | |
| --- | --- | --- | --- | --- | --- | --- | --- |
|  |  |  |  |  |  |  |  |
|  | **ZjbHLH** | **AtbHLH** | **PpbHLH** | **PbbHLH** | **MdbHLH** | **VvbHLH** | **GobHLH** |
| **I** | **15** | **22** | **23** | **40** | **42** | **17** | **33** |
| **II** | **13** | **13** | **3** | **19** | **17** | **15** | **19** |
| **III** | **1** | **5** | **1** | **6** | **4** | **1** | **8** |
| **IV** | **14** | **22** | **19** | **20** | **27** | **19** | **31** |
| **V** | **8** | **9** | **8** | **16** | **15** | **9** | **15** |
| **VI** | **5** | **9** | **2** | **14** | **13** | **0** | **15** |
| **VII** | **2** | **3** | **0** | **1** | **1** | **0** | **2** |
| **VIII** | **2** | **6** | **0** | **3** | **0** | **0** | **12** |
| **IX** | **1** | **2** | **0** | **1** | **1** | **1** | **0** |
| **X** | **1** | **3** | **0** | **1** | **1** | **1** | **0** |
| **XI** | **2** | **17** | **7** | **13** | **13** | **7** | **14** |
| **XII** | **13** | **17** | **12** | **22** | **19** | **10** | **34** |
| **XIII** | **3** | **5** | **4** | **8** | **3** | **5** | **11** |
| **XIV** | **3** | **6** | **5** | **10** | **10** | **5** | **5** |
| **XV** | **8** | **14** | **11** | **21** | **21** | **4** | **18** |
| **XVI** | **1** | **2** | **0** | **2** | **2** | **0** | **5** |
| **Total** | **92** | **155** | **94** | **197** | **189** | **94** | **222** |
