# Supplementary material for: Genome-wide analysis of the bHLH gene family in Chinese jujube (Ziziphus jujuba Mill.) and wild jujube
Source: BMC Genomics. 2019 Jul 10;20:568. doi: 10.1186/s12864-019-5936-2 (PMC6617894; doi:10.1186/s12864-019-5936-2)
Supplement: Supplementary file 8 — Table S3. The primers of ZjbHLH genes used in this study. (DOC 142 kb) [file 12864_2019_5936_MOESM8_ESM.doc]

| **Number** | **Gene Name** |  | **Sequence (5'-3')** | **Length(bp)** | **Product size(bp)** |
| --- | --- | --- | --- | --- | --- |
| 1 | ***ZjbHLH1*** | Forward | AGCGGCCCAAGGTTGATCG | 19 | 278 |
|  |  | Reverse | GGCATCTGCAGCATTCCTCATC | 22 |  |
| 2 | ***ZjbHLH2*** | Forward | TAGCGGTGTCACTGTTGGAG | 20 | 268 |
|  |  | Reverse | GAAGCTTGTGGTAGGCGTCA | 20 |  |
| 3 | ***ZjbHLH3*** | Forward | CAGCTCCAATGCAAACAGTGC | 21 | 281 |
|  |  | Reverse | AGCTTGTGCTAGGTGTCAGTG | 21 |  |
| 4 | ***ZjbHLH4*** | Forward | GGCAAGAAGAAAGGGTTGCC | 20 | 269 |
|  |  | Reverse | AGGGTCGGAGTAGTTGGTGT | 20 |  |
| 5 | ***ZjbHLH5*** | Forward | GCAAAGAAGATTCAGGGCCAA | 21 | 286 |
|  |  | Reverse | TTGGCGTGTTTCTGACTAGAAG | 22 |  |
| 6 | ***ZjbHLH8*** | Forward | AGAGTGTGGCATTGTAGCACAG | 22 | 293 |
|  |  | Reverse | TCTTAATGTCGCCTTTGCTCTT | 22 |  |
| 7 | ***ZjbHLH11*** | Forward | CTTGATGAGCGGAAACCACG | 20 | 278 |
|  |  | Reverse | ATGTCTTGCACCGACAATGG | 20 |  |
| 8 | ***ZjbHLH12*** | Forward | GCCTGCCAATGGAAGAGAAG | 20 | 229 |
|  |  | Reverse | TGCATCTCTCGAAGAACTCCC | 21 |  |
| 9 | ***ZjbHLH13*** | Forward | GCAAAGGCACTCCGCAAG | 18 | 241 |
|  |  | Reverse | CTGGATCTCGACCGTTGGTG | 20 |  |
| 10 | ***ZjbHLH14*** | Forward | ATGGAGTTTGGAGACCGGAG | 20 | 275 |
|  |  | Reverse | ATCGGATGTTCTTTCCGTGGT | 21 |  |
| 11 | ***ZjbHLH15*** | Forward | CCAGACGATCTCTCTGATGCTG | 22 | 269 |
|  |  | Reverse | CGCTGAAGGAGATTCAGGTC | 20 |  |
| 12 | ***ZjbHLH17*** | Forward | GTCGGACGGCTATACATGCTC | 21 | 257 |
|  |  | Reverse | TTCATCATCACCGGAAACCAG | 21 |  |
| 13 | ***ZjbHLH18*** | Forward | AGGAGCCAAGAGGGTCTGT | 19 | 278 |
|  |  | Reverse | CACCATCTGCAAAGAGTTGCG | 21 |  |
| 14 | ***ZjbHLH19*** | Forward | ACACAACCAGTACTACCAAGCC | 22 | 283 |
|  |  | Reverse | ATGTGGAAGCCACTCTTCCTTG | 22 |  |
| 15 | ***ZjbHLH21*** | Forward | TCTAGAACTCCTTCACATGCTC | 22 | 227 |
|  |  | Reverse | ACGAATACCACTGATTCCACAG | 22 |  |
| 16 | ***ZjbHLH23*** | Forward | TGATACGTGTGGTTCATCTAGCTC | 24 | 249 |
|  |  | Reverse | AGCTCATTCTTCTCCGCCTTC | 21 |  |
| 17 | ***ZjbHLH24*** | Forward | CGGCAGAGGTCTAAATCATGTAGTG | 25 | 265 |
|  |  | Reverse | CACGAAGCTCATTCTTCTCAGC | 22 |  |
| 18 | ***ZjbHLH29*** | Forward | ATTGGTGAAGGAAGGGACGGG | 21 | 286 |
|  |  | Reverse | TATCAACGTCTCCATTCGCTCC | 22 |  |
| 19 | ***ZjbHLH30*** | Forward | GGAAGAGATCGAGAACCAGAGG | 22 | 237 |
|  |  | Reverse | GCGAAGCTCTTGTCTTGTTG | 20 |  |
| 20 | ***ZjbHLH31*** | Forward | AAAGAGACGGCGTACGAGAAG | 21 | 258 |
|  |  | Reverse | TTGGAATCCGATTGCTGGTTTC | 22 |  |
| 21 | ***ZjbHLH34*** | Forward | ACCAGTGAAGAAGTTGAGAGCC | 22 | 185 |
|  |  | Reverse | AGCAGCTGTTCTAGTTCCCTC | 21 |  |
| 22 | ***ZjbHLH45*** | Forward | CTAAAGCTCTTGCCGCTTCT | 20 | 202 |
|  |  | Reverse | GGCACTGGACTTGTTTCTGC | 20 |  |
| 23 | ***ZjbHLH49*** | Forward | AACCCCAACAGCAAAGATGG | 20 | 257 |
|  |  | Reverse | AACTTTGTCGGCTCTGGACTC | 21 |  |
| 24 | ***ZjbHLH53*** | Forward | CGAAACGCCTTCACCTTTACC | 21 | 214 |
|  |  | Reverse | CTGTAGTGGATGGATTGGAGC | 21 |  |
| 25 | ***ZjbHLH54*** | Forward | TCGAAATGAAACAGCATCGCC | 21 | 209 |
|  |  | Reverse | CTGGTACAGATATGTGGGCAT | 21 |  |
| 26 | ***ZjbHLH60*** | Forward | TGTCTAGCCGAAGGTCGAGG | 20 | 214 |
|  |  | Reverse | AACAACTGGGACAGTCGCTC | 20 |  |
| 27 | ***ZjbHLH62*** | Forward | AGTACGGCACGCACTTATGG | 20 | 291 |
|  |  | Reverse | ATTCCCGACTCCAAGCTTCTG | 21 |  |
| 28 | ***ZjbHLH63*** | Forward | AGAGAAGCAGAGCAGCTGAAG | 21 | 207 |
|  |  | Reverse | GTGGCATATACAAACCAGCTCC | 22 |  |
| 29 | ***ZjbHLH65*** | Forward | CAACAACAGGGAATCTTCTGCT | 22 | 295 |
|  |  | Reverse | CACAGCAGCTAGTTTCATGGAC | 22 |  |
| 30 | ***ZjbHLH78*** | Forward | ATGGACAACCGATTCCCACC | 20 | 272 |
|  |  | Reverse | TCCGCCCAATCTACTCATGC | 20 |  |
| 31 | ***ZjbHLH79*** | Forward | ATAGTCAATCCACGGCTGGGC | 21 | 296 |
|  |  | Reverse | TACAAGTTGAGCCACTGCTCC | 21 |  |
| 32 | ***ZjbHLH81*** | Forward | ACTTGCAGTTCCAAGACTCTG | 21 | 253 |
|  |  | Reverse | GAACACGTACACTTGGCACG | 20 |  |
| 33 | ***ZjbHLH83*** | Forward | ACAGAAGCTCTTGCAGTTTCA | 21 | 262 |
|  |  | Reverse | TGAACACGAGCACTTGGACT | 20 |  |
| 34 | ***ZjbHLH87*** | Forward | GAATCCATCAAACCGCCCAAG | 21 | 238 |
|  |  | Reverse | TTGTTGGTCTATTAGCACACGC | 22 |  |
| 35 | ***ZjbHLH88*** | Forward | TACTGATCCACAAAGTGTCGCA | 22 | 181 |
|  |  | Reverse | GTTGATCATGGTTTGGTGAAGC | 22 |  |
| 36 | ***ZjbHLH90*** | Forward | ACTTCCGACACCAAGCCATC | 20 | 263 |
|  |  | Reverse | GGAGCATACATCCACAGGTCC | 21 |  |
| 37 | ***ZjbHLH92*** | Forward | TGCCAACATCAGAAGTCCCG | 20 | 204 |
|  |  | Reverse | TCCAGTCCGATGGTTTCGG | 19 |  |
