# Supplementary material for: Genome-wide analysis of the bHLH gene family in Chinese jujube (Ziziphus jujuba Mill.) and wild jujube
Source: BMC Genomics. 2019 Jul 10;20:568. doi: 10.1186/s12864-019-5936-2 (PMC6617894; doi:10.1186/s12864-019-5936-2)
Supplement: Supplementary file 5 [file 12864_2019_5936_MOESM3_ESM.doc]

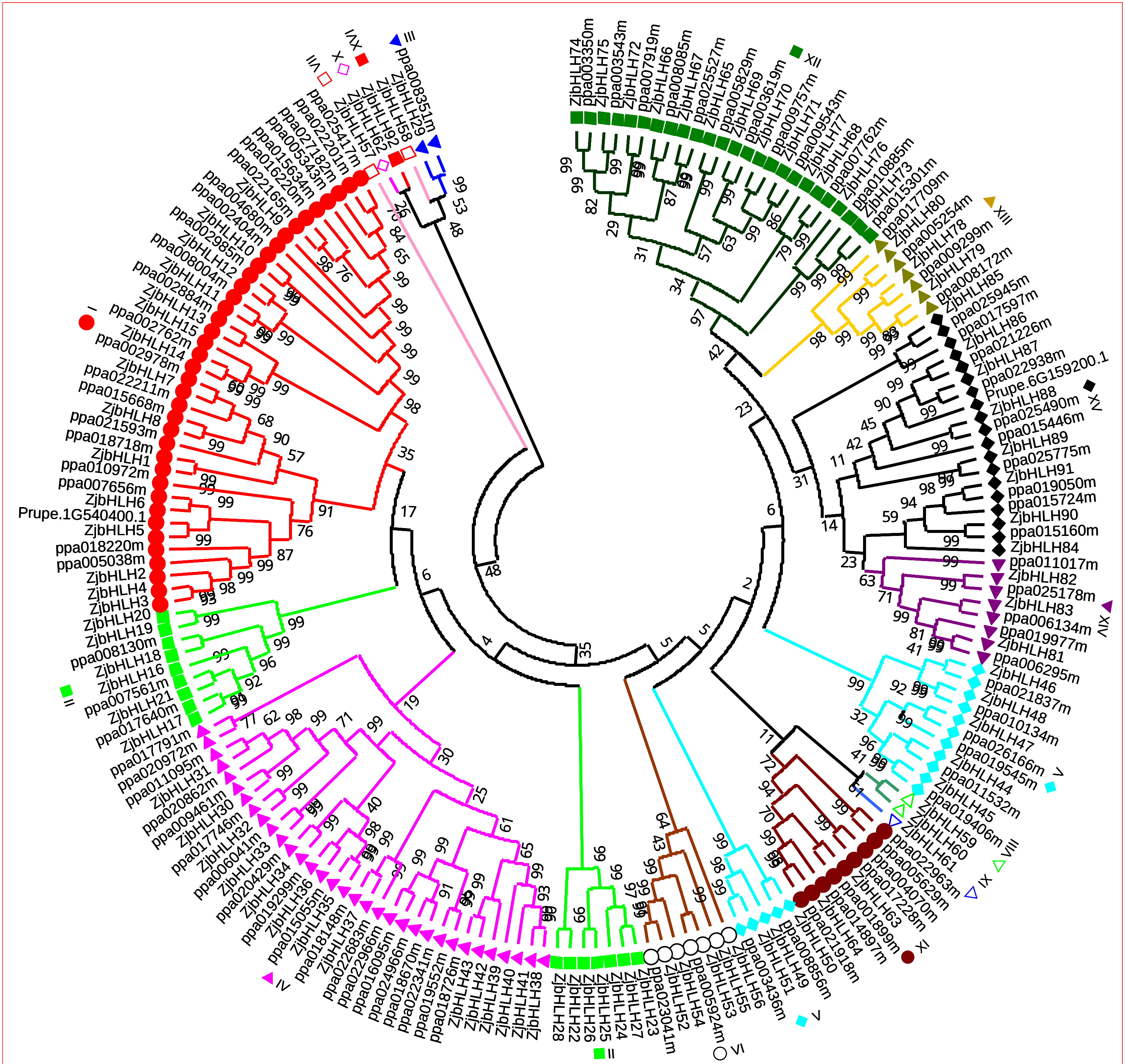
XII
ZjbHLH74
ppa003350m
ZjbHLH75
ppa003543m
ZjbHLH72
ppa007919m
ZjbHLH66
ppa008085m
ZjbHLH67
ppa025527m
ZjbHLH65
ppa005829m
ZjbHLH69
ppa003619m
ZjbHLH70
ppa009757m
ZjbHLH71
ppa009543m
ZjbHLH77
ZjbHLH68
ppa007762m
ZjbHLH76
ppa010885m
ZjbHLH73
ppa015301m
XIII
ppa017709m
ZjbHLH80
ppa005254m
ZjbHLH78
ppa009299m
ZjbHLH79
ppa008172m
ZjbHLH85
ppa025945m
ppa017597m
ZjbHLH86
ppa021226m
ZjbHLH87
ppa022938m
Prupe.6G159200.1
XV
ZjbHLH88
ppa025490m
ppa015446m
ZjbHLH89
ppa025775m
ZjbHLH91
ppa019050m
ppa015724m
ZjbHLH90
ppa015160m
ZjbHLH84
ppa011017m
ZjbHLH82
ppa025178m
ZjbHLH83
XIV
ppa006134m
ppa019977m
ZjbHLH81
ppa006295m
ZjbHLH46
ppa021837m
ZjbHLH48
ppa010134m
ZjbHLH47
ppa026166m
ppa019545m
V
ZjbHLH44
ppa011532m
ZjbHLH45
ppa019406m
VIII
ZjbHLH59
ZjbHLH60
IX
ZjbHLH61
ppa022963m
ppa005629m
ppa004070m
ppa017228m
ZjbHLH63
XI
ppa001899m
ppa014897m
ZjbHLH64
ppa021918m
ZjbHLH50
ppa008856m
ZjbHLH49
ZjbHLH51
V
ppa003436m
ZjbHLH56
ZjbHLH55
ZjbHLH53
ppa005924m
VI
ZjbHLH54
ZjbHLH52
ppa023041m
ZjbHLH23
ZjbHLH27
ZjbHLH24
ZjbHLH25
II
ZjbHLH26
ZjbHLH22
ZjbHLH28
ZjbHLH38
ZjbHLH41
ZjbHLH40
ZjbHLH39
ZjbHLH42
ZjbHLH43
ppa018726m
ppa019552m
ppa022341m
ppa018670m
ppa024966m
ppa016095m
ppa022966m
ppa022683m
ZjbHLH37
ppa018148m
IV
ZjbHLH35
ppa015055m
ZjbHLH36
ppa019299m
ZjbHLH34
ppa020429m
ZjbHLH33
ppa006041m
ZjbHLH32
ppa017746m
ZjbHLH30
ppa009461m
ppa020862m
ZjbHLH31
ppa011095m
ppa020972m
ppa017791m
ZjbHLH17
ppa017640m
ZjbHLH21
II
ppa007561m
ZjbHLH16
ZjbHLH18
ppa008130m
ZjbHLH19
ZjbHLH20
ZjbHLH3
ZjbHLH4
ZjbHLH2
ppa005038m
ppa018220m
ZjbHLH5
Prupe.1G540400.1
ZjbHLH6
ppa007656m
ppa010972m
ZjbHLH1
ppa018718m
ppa021593m
ZjbHLH8
ppa015668m
ppa022211m
ZjbHLH7
ppa002978m
I
ZjbHLH14
ppa002762m
ZjbHLH15
ZjbHLH13
ppa002884m
ZjbHLH11
ppa008004m
ZjbHLH12
ppa002985m
ZjbHLH10
ppa002404m
ppa004680m
ZjbHLH9
ppa022165m
ppa016220m
ppa015634m
ppa005343m
ppa027182m
ppa022201m
ppa025417m
ZjbHLH57
VII
ZjbHLH62
X
ZjbHLH92
XVI
ZjbHLH58
ZjbHLH29
ppa008351m
III
