# Supplementary material for: Genome-wide analysis of the bHLH gene family in Chinese jujube (Ziziphus jujuba Mill.) and wild jujube
Source: BMC Genomics. 2019 Jul 10;20:568. doi: 10.1186/s12864-019-5936-2 (PMC6617894; doi:10.1186/s12864-019-5936-2)
Supplement: Supplementary file 6 [file 12864_2019_5936_MOESM4_ESM.doc]

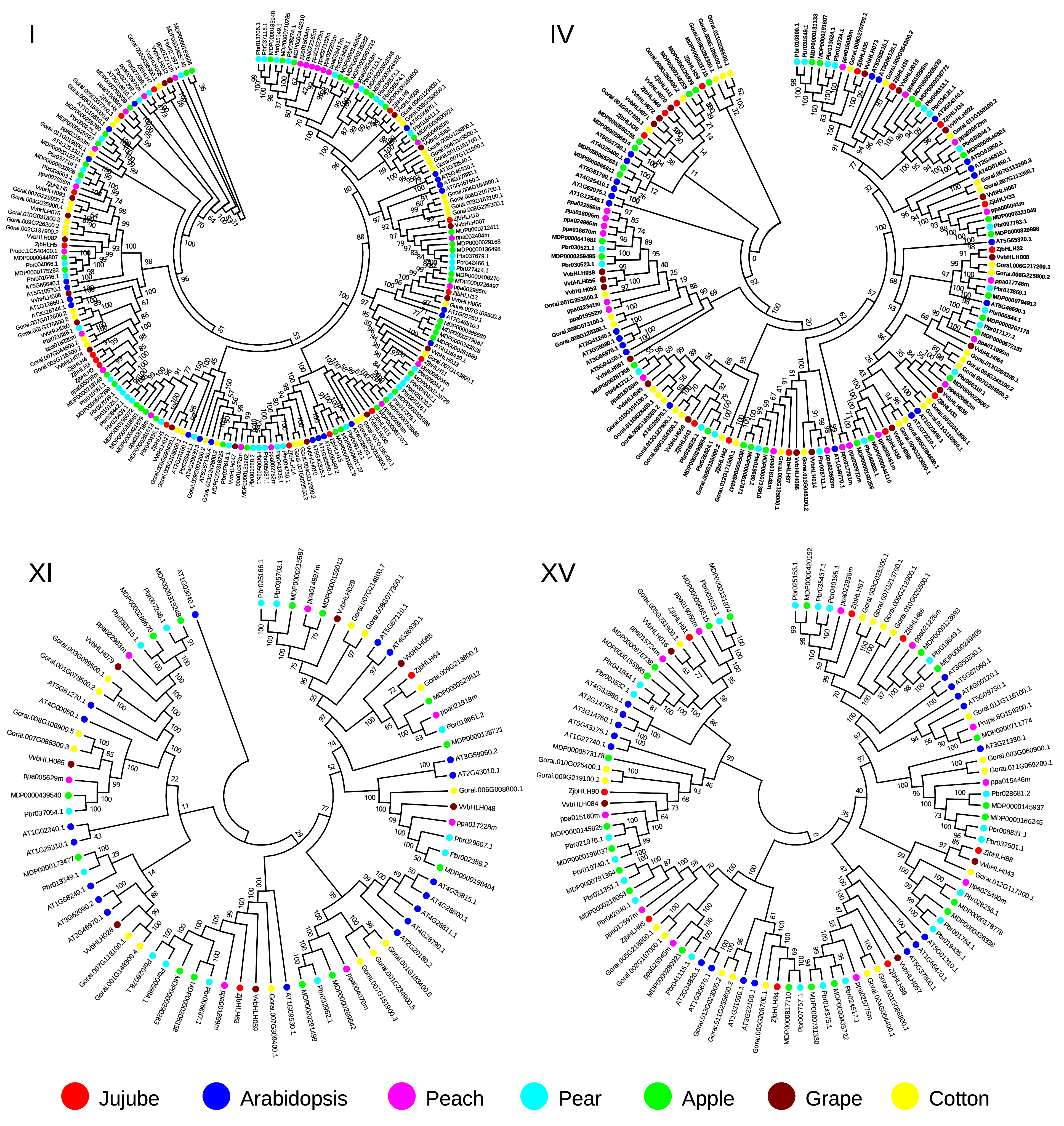
I
IV
XI
XV
Jujube
Arabidopsis
Peach
Pear
Apple
Grape
Cotton
